# Cure of Spina Bifida by Injection of Tincture of Iodine

**Published:** 1848-04

**Authors:** 


*On the Cure of Spina Bifida by Injection of the Tincture of Iodine.*—This disease and malformation not only constitute one of the most distressing affections to which infants are subject, being attended often with incontinence of the urine and feces, paralysis of the lower extremities and idiocy, but is also according to the best authorities, and indeed is shown by general experience to be incurable, with few and rare exceptions. For if in the annals of surgery there are to be found a certain number of successful cases, yet when considered with reference to the whole number occurring, it will be seen at once that no plan of treatment yet proposed affords more than feeble chances of success.

Of these methods of treatment, it is not a little singular that the one which consists in evacuating the fluid more or less perfectly should have been generally adopted, notwithstanding that the evacuating of serous fluid by puncture in every other situation is generally found to aggravate the disease. In this case it is liable to prove fatal by removing the necessary compression on the central organs of the nervous system. If the method has sometimes succeeded, it was from the inflammation induced by repeated punctures checking effusion and producing absorption, and not by any means from evacuating the fluid, which only gives it a greater tendency to increase. This danger and that which results from ulcerative inflammation at the point of puncture, on a tissue like the covering of this species of tumor should prohihit its performance. We were led to these views from reflecting on a case of Spina Bifida in the Chicago Hospital, in a girl said to be 13 years of age, in whom the tumor was situated at the top of the sacrum, being nine inches in circumference and about three inches in height, with thin walls. The girl had been paralytic in the lower members, but withing three years had acquired a partial use of them. She was idiotic, and passed both the feces and urine witout regard to place. From neglect of cleanliness, numerous ulcerations and large cicatrices had, from time to time, been formed upon the pelvis and thighs.

Under these almost hopeless circumstances I determined to inject into the sac a solution of Iodine, with a view of exciting inflammation and procuring absorption. This was done on the 2d Dec., 1847, in the followinng manner: A small puncture was made with the lancet on the sound skin about half an inch from the base of the tumor and a trochar of the size of a common knitting needle carried obliquely into the sac. Through the canula of this a solution of gr. j. of Hyd. Potass. with gr. ss. of Iodine in f. ℥i. of water, was thrown into the sac and the instrument witdrawn. A sharp pain followed which soon subsided. Compresses and a bandage were applied to prevent the escape of the fluid, and the child was laid in bed. There succeeded redness, heat and tension of the tumor with tenderness to the touch and some febrile symptoms, for which a cathartic was administered and evaporating lotions applied to the part. In the course of a week these symptoms subsided, and the tumor became soft, yielding, and diminished in size. Compression by means of a roller around the pelvis was then applied, and kept up with as great degree of force as could be borne, but the filthiness of the patient and her indocility prevented this from being applied with regularity. It was frequently removed for twelve hours or more at a time. Still it diminished, and on the 27th Dec. was about half its former size.

At this time a second injection was used of half the strength of the first. This produced but little heat or pain and the compression was continued. On the 15th Jan. 1848, the fluid was so far absorbed as to render it easy to press it down almost to a level with the surrounding skin. A spring truss was then substituted, and at the present time the sac lies in wrinkles, the bony opening can be distinctly felt, and there is no increase of swelling when the pressure is removed. Recently there has been manifested a decided improvement in the intellect of the child, the other difficulties remained the same, but with the removal of the cause the partial paralysis will doubtless gradually disappear. The retention of the natural evacuations must depend upon the development of the intelligence and the gaining of a control over the voluntary muscles. In its present condition, this case shows that the injection of a solution of iodine, followed by suitable treatment, is capable of curing an ancient case of Hydrorachitis, and (so far as a single case can be taken as a guide,) with but little danger. Further experience will be required to determine the strength and quantity of the medicine to be used. the frequency of the repetitions; in younger subjects than this, it is obvious that the dose employed should be not more than a fourth of that used at first in this case.—To those who would deem such a proceeding extremely hazardous, we would remark that a number of facts have within the last few years come under our observation, tending to show that serous membranes are, when filled with serum, much lsss disposed to inflammation than when in a healthy state. Experiments are required to show which among the astringent and stimulating fluids can be brought in contact with them with least danger, meanwhile the safety with which solutions of Iodine may be thrown into the large articulations, into the sac of a congenital hernia, and in some instances into the peritoneum, and in this case into the arachnoid membrane, will free us from the charge of presumption in suggesting that it, or some other solution, may be worthy of trial in those cases of Hydrocephalus, ascites and hydrothorax, which are hopeless under ordinary treatment.—*Illinois Med. Journal.*

